# Signature of long-lived memory CD8^+^ T cells in acute SARS-CoV-2 infection

**DOI:** 10.1038/s41586-021-04280-x

**Published:** 2021-12-07

**Authors:** Sarah Adamo, Jan Michler, Yves Zurbuchen, Carlo Cervia, Patrick Taeschler, Miro E. Raeber, Simona Baghai Sain, Jakob Nilsson, Andreas E. Moor, Onur Boyman

**Affiliations:** 1 Department of Immunology, University Hospital Zurich, Zurich, Switzerland; 2 Department of Biosystems Science and Engineering, ETH Zurich, Basel, Switzerland; 3 Faculty of Medicine, University of Zurich, Zurich, Switzerland

**Keywords:** Immunological memory, Infection

## Abstract

Immunological memory is a hallmark of adaptive immunity and facilitates an accelerated and enhanced immune response upon re-infection with the same pathogen^1,2^. Since the outbreak of the ongoing COVID-19 pandemic, a key question has focused on which SARS-CoV-2-specific T cells stimulated during acute infection give rise to long-lived memory T cells^3^. Here, using spectral flow cytometry combined with cellular indexing of transcriptomes and T cell receptor sequencing, we longitudinally characterized individual SARS-CoV-2-specific CD8^+^ T cells of patients with COVID-19 from acute infection to 1 year into recovery and found a distinct signature identifying long-lived memory CD8^+^ T cells. SARS-CoV-2-specific memory CD8^+^ T cells persisting 1 year after acute infection express CD45RA, IL-7 receptor-α and T cell factor 1, but they maintain low expression of CCR7, thus resembling CD45RA^+^ effector memory T cells. Tracking individual clones of SARS-CoV-2-specific CD8^+^ T cells, we reveal that an interferon signature marks clones that give rise to long-lived cells, whereas prolonged proliferation and mechanistic target of rapamycin signalling are associated with clonal disappearance from the blood. Collectively, we describe a transcriptional signature that marks long-lived, circulating human memory CD8^+^ T cells following an acute viral infection.

## Abstract (web summary)

Evidence of a transcriptional signature that marks precursors of long-lived CD8^+^ memory T cells in SARS-CoV-2 infection.

## Main

The coronavirus disease 2019 (COVID-19) pandemic has taken an extraordinary toll on global health and economy, affecting billions of lives all over the world. The ongoing vaccination efforts appear to curtail the spread of severe acute respiratory syndrome coronavirus 2 (SARS-CoV-2) and prevent severe disease, even as new virus variants emerge^4,5^. Yet, prevailing questions concern whether and how exposure to SARS-CoV-2 by infection or immunization might result in long-term protective immunity.

On encountering their cognate antigen on antigen-presenting cells, antigen-specific CD8^+^ T cells proliferate and differentiate into effector cells aimed at controlling the pathogen by killing virus-infected host cells. Following virus elimination, 90–95% of effector T cells undergo apoptosis, whereas some antigen-specific T cells survive to become long-lived memory T cells that are able to protect the host from re-infection with the same pathogen^2,6^.

While antigen-specific effector T cell responses are generated during acute SARS-CoV-2 infection^7–12^ and persist for several months^13–17^, little is known about changes in memory phenotypes over time. Previous studies using live-attenuated virus vaccines in healthy donors^18–21^ have described phenotypical trajectories of human antigen-specific T cell populations. However, it is unknown whether infection with a natural virus generates comparable memory T cell responses in humans, as infection route, viral load, inflammation and various host-related factors are likely to affect T cell responses and memory formation. Moreover, phenotypical and transcriptional trajectories at the single T cell receptor (TCR) level and the factors instructing individual effector T cell clones on their development to long-lived memory T cells have not been investigated in humans.

### Phenotype of SARS-CoV-2^+^ CD8^+^ T cells

**Fig. 1:**
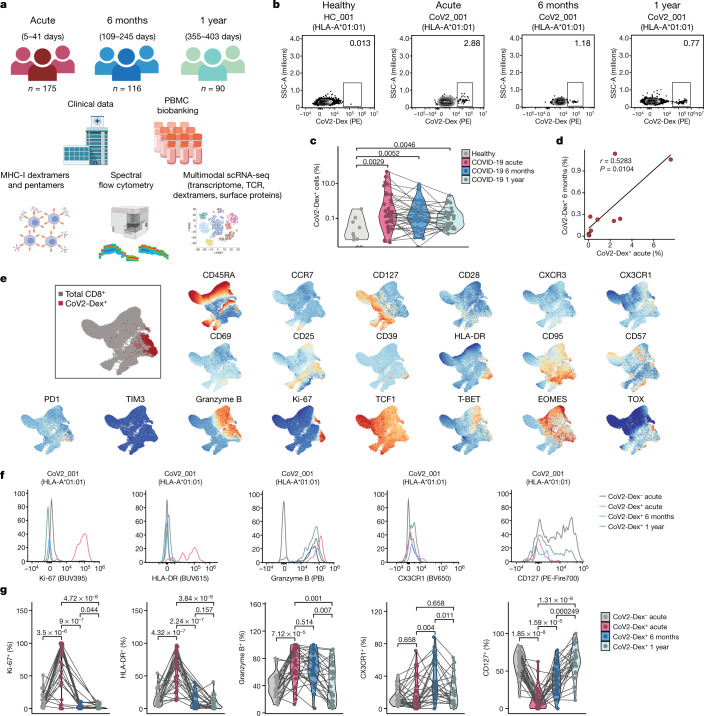
Characteristics of antigen-specific CD8^+^ T cells during acute and memory phases of SARS-CoV-2 infection. **a**, Overview of study design. PBMC, peripheral blood mononuclear cell. **b**, Representative plots of CoV2-Dex staining. PE, Phycoerythrin. Numbers in the plots indicate percentage of parent population. **c**, Frequency of CoV2-Dex^+^ cells in healthy donors and patients with COVID-19 during acute infection and 6 months and 1 year after infection. Each dot represents an independent donor at the indicated timepoint (*n* = 10 healthy, *n* = 37 acute, *n* = 32 6 months, *n* = 29 1 year after infection). *P* values are shown. **d**, Linear regression of frequency of CoV2-Dex^+^ cells 6 months after infection as a function of CoV2-Dex^+^ cell frequencies during acute infection (*n* = 11). The *P* value was calculated with *t*-statistic. **e**, Uniform manifold approximation and projection (UMAP) plots of marker expression for up to 2,000 CD8^+^ T cells from each sample collected during acute infection (*n* = 37) analysed by spectral flow cytometry. Regions with high marker expression appear in red. An overlay of CoV2-Dex^+^ cells (red) and total CD8^+^ T cells (grey) is shown in the top left. **f**, Representative histograms showing expression of selected markers on CoV2-Dex^–^ and CoV2-Dex^+^ cells. **g**, Frequency of Ki-67^+^, HLA-DR^+^, granzyme B^+^, CX3CR1^+^ and CD127^+^ cells in CoV2-Dex^–^ (grey) and CoV2-Dex^+^ cells during acute infection and 6 months and 1 year after infection. Analysis was conducted on paired samples from acute infection versus 6 months and/or 1 year after infection (*n* = 28 acute, *n* = 24 6 months, *n* = 29 1 year). The grey lines connect individual donors sampled at different timepoints. *P* values were calculated using a Wilcoxon–Mann–Whitney test in **c** and **g** and corrected for multiple comparisons in **g**. All tests were performed two-sided.

**Extended Data Fig. 1:**
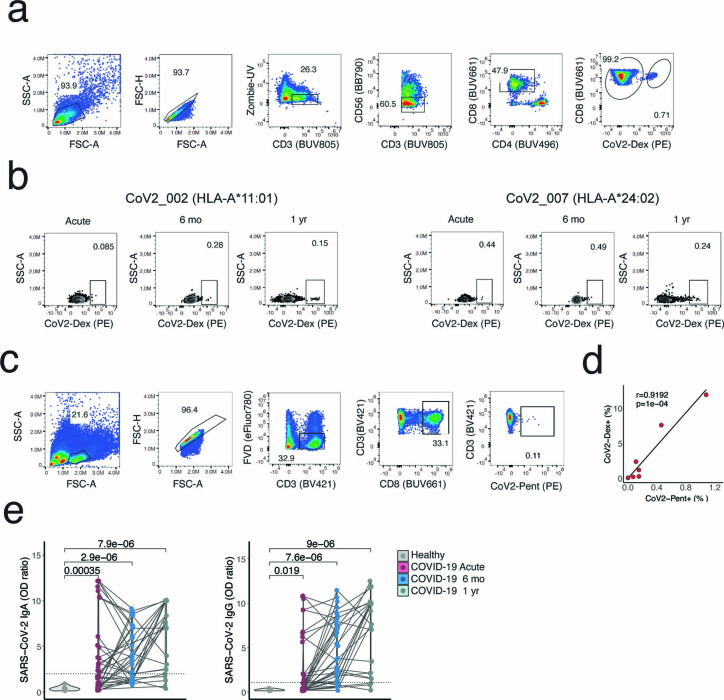
Gating strategy for antigen-specific CD8^+^ T cells and SARS-CoV2-specific antibodies in healthy donors and COVID-19 patients. **a**, Gating strategy for CoV2-Dex^+^ cells. **b**, Representative plot of CoV2-Dex staining for HLA-A*11:01 and HLA-A*24:02. **c**, Gating strategy for CoV2-Pent^+^ cells. **d**, Linear regression of frequency of CoV2-Dex^+^ cells as a function of frequency of CoV2-Pent^+^ cells (*n* = 7). The p-value was calculated with t-statistic. **e**, SARS-CoV2-specific IgA (left) and IgG (right) of healthy donors compared to COVID-19 patients during acute infection, six months and one year after infection (*n* = 13 healthy *, n* = 46 acute, *n* = 41 six months, *n* = 30 one year). Dashed lines represent diagnostic cut-off values, i.e., 2.0 and 1.1 for IgA and IgG, respectively. P-values were calculated with a Mann-Whitney-Wilcoxon test. All tests were performed two-sided.

To assess the dynamics of antigen-specific T cells in COVID-19, we recruited 175 patients with real-time PCR (RT–PCR)-confirmed COVID-19, sampled during their symptomatic acute phase and followed up 6 months and 1 year after acute infection (Fig. 1a). We conducted human leukocyte antigen (HLA) typing on all patients and healthy controls and selected individuals carrying the HLA-A*01:01, HLA-A*11:01 or HLA-A*24:02 alleles for this study (*n* = 47 patients and *n* = 13 healthy controls; characteristics are included in Extended Data Table 1). In these individuals, SARS-CoV-2-specific CD8^+^ T cells were detected by using HLA-A*01:01, HLA-A*11:01 and HLA-A*24:02 major histocompatibility complex class I (MHC-I) dextramers^12^, hereafter termed CoV2-Dex (Fig. 1b, Extended Data Fig. 1a, b), and validated by using HLA-A*01:01 and HLA-A*11:01 MHC-I pentamers^22^, hereafter termed CoV2-Pent (Extended Data Fig. 1c, d). Healthy controls were seronegative for SARS-CoV-2 spike-specific IgG and IgA (Extended Data Fig. 1e).

**Extended Data Fig. 2:**
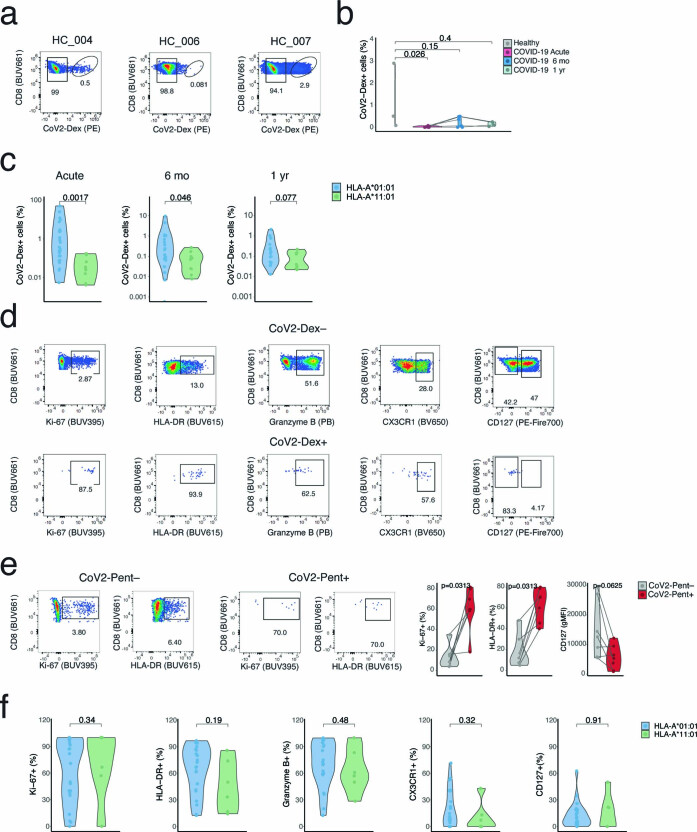
HLA-A*24:02 dextramer staining and CoV2-Dex^+^ cell frequency and phenotype based on HLA. **a**, Representative plots of CoV2-Dex (HLA-A*24:02) staining in healthy donors. **b**, Percentage of CoV2-Dex^+^ cells in HLA-A*24:02 healthy donors and COVID-19 patients in acute infection and six months and one year after infection. Dots represent independent donors at each timepoint (*n* = 3 healthy, *n* = 6 acute, *n* = 5 six months, *n* = 3 one year). Gray lines connect individual donors sampled at different timepoints. **c**, Percentage of CoV2-Dex^+^ cells in HLA-A*01:01 and HLA-A*11:01 in acute infection and six months and one year after infection (HLA-A*01:01 *n* = 26 and HLA-A*11:01 *n* = 10 acute, HLA-A*01:01 *n* = 21 and HLA-A*11:01 *n* = 9 six months, HLA-A*01:01 *n* = 20 and HLA-A*11:01 *n* = 9 one year). **d**, Gating strategy for phenotypical analysis of CoV2-Dex^+^ compared to CoV2-Dex^–^ cells. **e**, Gating strategy for phenotypical analysis of CoV2-Pent^+^ compared to CoV2-Pent^–^ cells and frequency of Ki-67^+^, HLA-DR^+^ and CD127 levels in CoV2-Pent^–^ (gray) and CoV2-Pent^+^ (red) cells in acute COVID-19 (*n* = 7). **f**, Frequency of Ki-67^+^, HLA-DR^+^, granzyme B^+^, CX3CR1^+^ and CD127^+^ cells in CoV2-Dex^+^ cells in patients with an HLA-A*01:01 versus HLA-A*11:01 allele (HLA-A*01:01 *n* = 22 and HLA-A*11:01 *n* = 6) in the acute phase. **b**, **c**, and **f**, P-values were calculated with a Mann-Whitney-Wilcoxon test. **e**, P-values were calculated with a Wilcoxon signed-rank test. All tests were performed two-sided.

SARS-CoV-2-specific CD8^+^ T cells were found in most patients carrying an HLA-A*01:01 or HLA-A*11:01 allele during acute infection and 6 months later (Fig. 1c). Moreover, we detected SARS-CoV-2-specific CD8^+^ T cells 1 year after acute SARS-CoV-2 infection (Fig. 1b, c). Staining with HLA-A*24:02 CoV2-Dex (which carried a spike-derived peptide, QYIKWPWYI) showed much higher background staining in some healthy donors (Extended Data Fig. 2a), possibly due to cross-reactivity. In individuals carrying HLA-A*24:02 alleles, we did not observe increased frequencies of CoV2-Dex^+^ cells during acute infection compared to healthy donors (Extended Data Fig. 2b), contrary to patients with HLA-A*01:01 or HLA-A*11:01 alleles (Fig. 1c). This finding might indicate that T cells specific for this spike epitope did not undergo marked expansion during SARS-CoV-2 infection. We could not determine whether HLA-A*24:02 CoV2-Dex^+^ cells had an activated or proliferating phenotype due to low cell numbers. Furthermore, we noted a lower reactivity to HLA-A*11:01 dextramers than to HLA-A*01:01 dextramers (Extended Data Fig. 2c) during acute infection, which persisted 6 months after infection. These data suggest heterogeneity in effector and memory T cell responses based on HLA type and specific epitopes, although they need careful interpretation due to limited patient numbers.

In patients with an HLA-A*01:01 or HLA-A*11:01 allele, the frequency of CoV2-Dex^+^ cells during acute infection correlated with the frequency of specific cells at the memory phase (Fig. 1d). In acute infection, flow cytometry analysis of CoV2-Dex^+^ cells revealed a circumscript phenotype of activated cells, dominated by high abundance of Ki-67 and HLA-DR (Fig. 1e, f, Extended Data Fig. 2d, e). CoV2-Dex^+^ cells also expressed granzyme B and tended to have higher expression of the terminal differentiation marker CX3CR1, whereas surface CD127 (also known as IL-7 receptor-α) was markedly downregulated (Fig. 1f, g). At the 6-month and 1-year timepoints, frequencies of Ki-67^+^ and HLA-DR^+^ CoV2-Dex^+^ cells declined and the frequency of CD127^+^ cells increased (Fig. 1f, g), indicating a transition from effector to memory state^6,23^. We did not observe phenotypical differences between HLA-A*01:01 and HLA-A*11:01 (Extended Data Fig. 2f).

**Extended Data Fig. 3:**
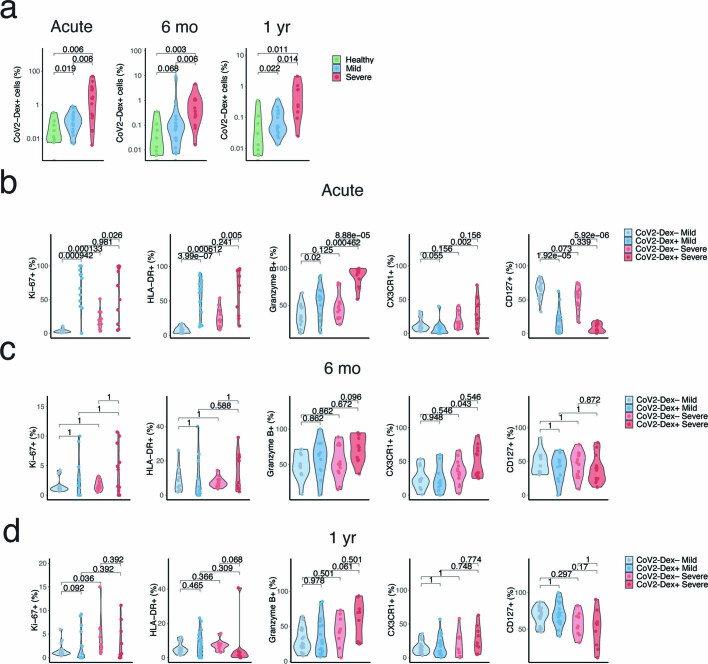
Frequency and phenotype of antigen-specific CD8^+^ T cells relative to disease severity. **a**, Frequency of CoV2-Dex^+^ CD8^+^ T cells in healthy controls and patients with mild and severe disease during acute COVID-19 six months and one year after infection (*n* = 10 healthy; acute: *n* = 21 mild, *n* = 15 severe; six months: *n* = 18 mild, *n* = 16 severe; one year: *n* = 19 mild, *n* = 10 severe). **b**–**d**, Frequency of Ki-67^+^, HLA-DR^+^, granzyme B^+^, CX3CR1^+^ and CD127^+^ in CoV-2Dex^–^ and CoV2-Dex^+^ cells in patients with mild versus severe disease (**b**) during acute infection, (**c**) six months and (**d**) one year after infection (acute: *n* = 16 mild, *n* = 12 severe; six months: *n* = 11 mild, *n* = 16 severe; one year: *n* = 19 mild, *n* = 10 severe). P-values were calculated with a Mann-Whitney-Wilcoxon test and adjusted for multiple comparisons using the Holm method. All tests were performed two-sided.

Notably, disease severity seemed to positively correlate with the extent of CD8^+^ T cell responses during acute infection, as well as frequencies of CoV2-Dex^+^ cells 6 months and 1 year after infection, although expansion of CoV2-Dex^+^ cells was also evident in patients with mild disease (Extended Data Fig. 3a). During acute infection, both proliferation and activation were only minimally affected by disease severity in the CoV2-Dex^+^ compartment, whereas a relevant difference was observed in CoV2-Dex^–^ cells (Extended Data Fig. 3b). This discrepancy could be due to higher bystander activation in severe disease or higher abundance of undetected SARS-CoV-2-reactive T cells. Patients with severe disease showed higher expression of granzyme B and CX3CR1 on CoV2-Dex^+^ cells, possibly indicating a different T cell differentiation program during the acute phase of severe COVID-19. These differences were no longer evident 6 months and 1 year after infection (Extended Data Fig. 3c, d).

### Transcriptome of SARS-CoV-2^+^ CD8^+^ clones

**Fig. 2:**
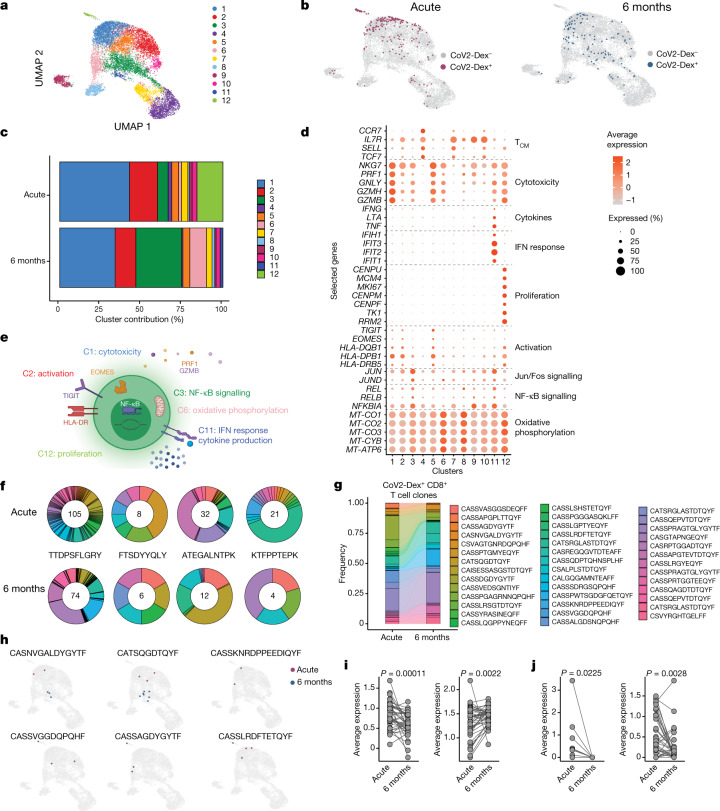
Transcriptional makeup of SARS-CoV-2-specific CD8^+^ T cell clones. **a**, Single-cell transcriptomes of CD8^+^ T cells displayed by UMAP. Seurat-based clustering of 14,853 cells, coloured based on cluster ID. **b**, UMAP as in **a**; CoV2-Dex^+^ cells from the acute infection (red) and 6 months after infection (blue) are highlighted. **c**, Cluster composition of CoV2-Dex^+^CD8^+^ T cells in acute infection versus 6 months after infection. **d**, Average expression (colour scale) and the percentage of expressing cells (size scale) of selected genes in indicated clusters. **e**, Schematic summary of the main clusters differentially represented in acute infection and 6 months after infection. **f**, Clonotype distribution in CoV2-Dex^+^ T cell clones (at least one CoV2-Dex^+^ cell per clone) for each of the four epitopes assessed. The number of T cell clones specific for the indicated epitopes at acute infection (top) and 6 months after infection (bottom) is provided within the circle. **g**, Alluvial plot showing relative representation of single clones present during acute infection and 6 months after infection (*n* = 41). **h**, UMAP as in **a**; cells from individual CoV2-Dex^+^CD8^+^ T cell clones in acute infection (red) and 6 months after infection (blue) are highlighted. **i**, Gene signature scores of individual CD8^+^ T cell clones in acute infection (acute gene signature, left) versus 6 months after infection (recovery gene signature, right) (*n* = 41). **j**, Expression of *MKI67* (left) and *HLA-DRB5* (right) in individual CD8^+^ T cell clones in acute infection versus 6 months after infection (*n* = 41). *P* values were calculated using a Wilcoxon signed-rank test in **i** and **j**.

**Extended Data Fig. 4:**
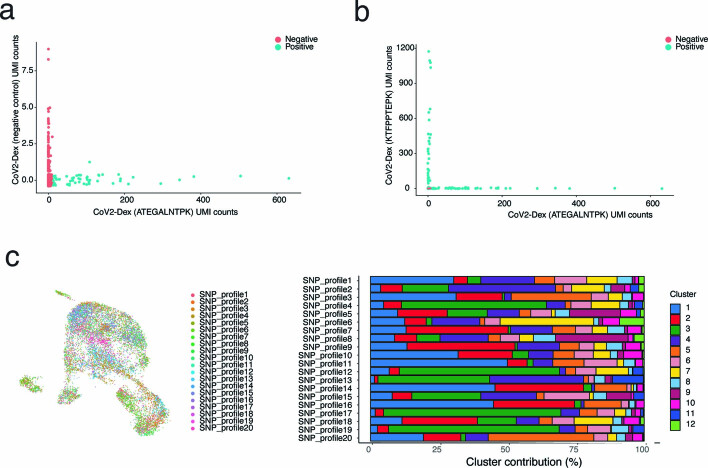
Definition of CoV2-Dex^+^ cells and single patient contribution to individual clusters. **a**, Unique molecular identifier (UMI) counts for CoV2-Dex HLA-A*11:01 (ATEGALNTPK) versus UMI counts for negative control dextramer; cells defined as CoV2-Dex HLA-A*11:01 (ATEGALNTPK)^+^ are depicted in blue. **b**, (UMI) counts for CoV2-Dex HLA-A*11:01 (ATEGALNTPK) versus UMI counts for CoV2-Dex HLA-A*11:01 (KTFPPTEPK). **c**, Uniform manifold approximation and projection (UMAP) plot colored by patient ID (left) and cluster distribution for single patients (right).

**Extended Data Fig. 5:**
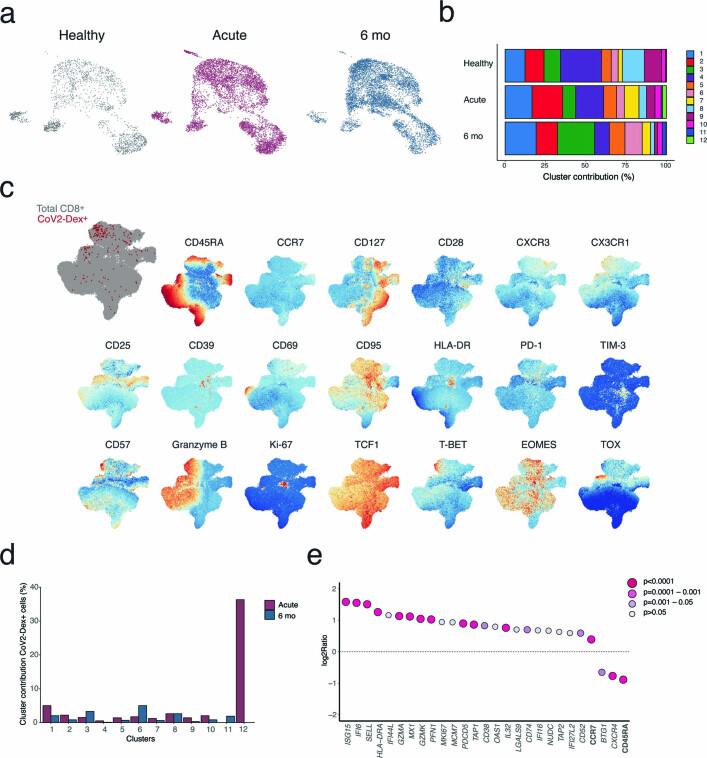
Characteristics of antigen-specific CD8^+^ T cells six months after infection. **a**, UMAP plot of transcriptomes from CD8^+^ T cells of healthy individuals (gray), COVID-19 patients in acute infection (red), and six months after infection (blue). **b**, Cluster contribution to total CD8^+^ T cells of healthy subjects, acute infection, and six months after infection. **c**, UMAP plots of marker expression for up to 2,000 CD8^+^ T cells from each sample collected six months after infection analyzed by spectral flow cytometry. Regions with high expression of specific markers appear red. Overlay of CoV2-Dex^+^ cells (red) and total CD8^+^ T cells (gray) is shown on the upper left. **d**, Percentages of CoV2-Dex^+^ cells contributing to indicated clusters during acute infection or six months after infection. Percentages are calculated on total cells per cluster per timepoint. **e**, Expression of selected genes (italicized), as well as CCR7 and CD45RA proteins determined by Totalseq^TM^, for CoV2-Dex^+^ cells detected in the acute versus recovery phase. P-values were calculated using a Mann-Whitney-Wilcoxon test and a Bonferroni correction was applied for multiple comparisons.

To examine the transcriptional phenotype of individual SARS-CoV-2-specific CD8^+^ T cells, we sorted CoV2-Dex^+^CD8^+^ T cells and CoV2-Dex^–^CD8^+^ T cells, mixed them at a 1:10 ratio, and performed single-cell RNA sequencing (scRNA-seq) on a subgroup of patients (*n* = 20 acute and *n* = 19 6-month timepoint). We classified sequenced cells as CoV2-Dex^–^ or CoV2-Dex^+^ based on their dCODE Dextramer unique molecular identifier counts (Methods, Extended Data Fig. 4a) and positivity for a single SARS-CoV-2 epitope (Extended Data Fig. 4b). Unbiased clustering revealed 12 distinct CD8^+^ T cell clusters (Fig. 2a), none of which was dominated by a single patient (Extended Data Fig. 4c). Some clusters showed nearly complete segregation between the acute and memory phases (Extended Data Fig. 5a, b). In line with our flow cytometry data (Fig. 1e, Extended Data Fig. 5c), CoV2-Dex^+^CD8^+^ T cells showed a rather segregated transcriptional makeup during acute infection, whereas their transcriptional state was more heterogeneous 6 months after infection (Fig. 2b). Comparing the contribution of CoV2-Dex^+^ cells to different clusters, we observed that clusters 1, 2 and 12 dominated the CoV2-Dex^+^CD8^+^ T cell response in the acute phase, whereas clusters 3, 6 and 11 became prominent in the recovery phase (Fig. 2c, Extended Data Fig. 5d). While clusters 1, 2 and 12 corresponded to cytotoxic, activated and proliferating cells, respectively, cluster 3 showed a signature marked by enrichment of NF-κB and Jun/Fos signalling, cluster 6 displayed an oxidative phosphorylation signature, and cluster 11 showed a dual signature marked by enrichment of interferon (IFN) response genes and genes encoding the effector cytokines IFNγ, tumour necrosis factor (TNF) and lymphotoxin-α (LTα) (Fig. 2d, e). Similarly, among genes with significantly higher expression in CoV2-Dex^+^ cells from the acute phase versus the recovery phase, we found genes related to cytotoxicity (*GZMA*, *GZMK* and *PFN1*), activation (*HLA-DRA*, *CD38* and *PDCD5*) and proliferation (*MKI67*, *MCM7* and *NUDC1*), along with IFN response genes (*IFI6*, *MX1*, *IFI27L2* and *IFI44L*) (Extended Data Fig. 5e). *SELL* (which encodes CD62L) appeared to be enriched in cells retrieved during the acute phase rather than the recovery phase (Extended Data Fig. 5e).

**Extended Data Fig. 6:**
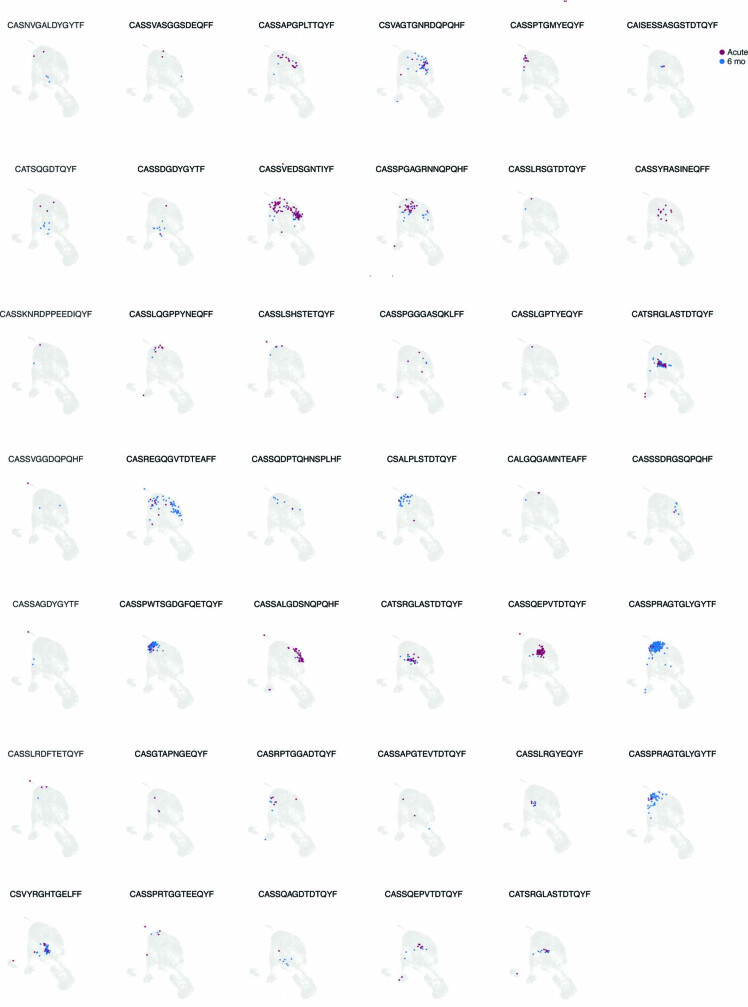
Transcriptome of SARS-CoV-2-specific CD8^+^ T cell clones. UMAP plots highlighting single-cell transcriptomes belonging to individual CoV2-Dex^+^ T cell clones in acute infection (red) and six months after infection (blue).

To identify phenotypical trajectories in individual antigen-specific T cell clones, we performed TCR sequencing of CoV2-Dex^+^ cells, which revealed several antigen-specific CD8^+^ T cell clones for each epitope investigated (Fig. 2f). Clones were considered antigen-specific if any of the clonal cells were CoV2-Dex^+^ (data available as Supplementary Dataset 1), and clones that were CoV2-Dex^+^ in the acute phase were considered CoV2-Dex^+^ independently of CoV2-Dex staining at six months after infection, and vice versa. The number of clones detected during convalescence was markedly lower than that detected during the acute phase of infection (Fig. 2f). In most cases, but not all, dominant clones in the acute phase corresponded to the largest clones found in the recovery phase (Fig. 2g). The phenotypical changes in acute infection versus the recovery phase on the general CoV2-Dex^+^ population were reflected in individual T cell clones. Thus, analysis of individual CoV2-Dex^+^ clones showed multiple clones containing cells from clusters 1, 2 or 12 during acute infection and cells from clusters 3, 6 and 11 during recovery (Fig. 2h, Extended Data Fig. 6). To better compare gene expression in acute infection versus recovery across all clones, we compiled an ‘acute gene signature’ comprising *NKG7*, *PRF1*, *GZMB*, *CENPU*, *CENPF* and *MKI67*, and a ‘recovery gene signature’ comprising *TNF*, *IFIT2*, *IFIT3*, *MT-CO1*, *MT-CO2* and *MT-ATP6*. We observed a significant decrease in acute gene signature transcripts in individual T cell clones from the acute phase to the recovery phase, which was paralleled by an increase in the recovery gene signature (Fig. 2i). Accordingly, individual T cell clones showed a decrease in *MKI67* and *HLA-DRB5* expression between the acute phase and the recovery phase (Fig. 2j).

### Memory paths of SARS-CoV-2^+^CD8^+^ cells

**Fig. 3:**
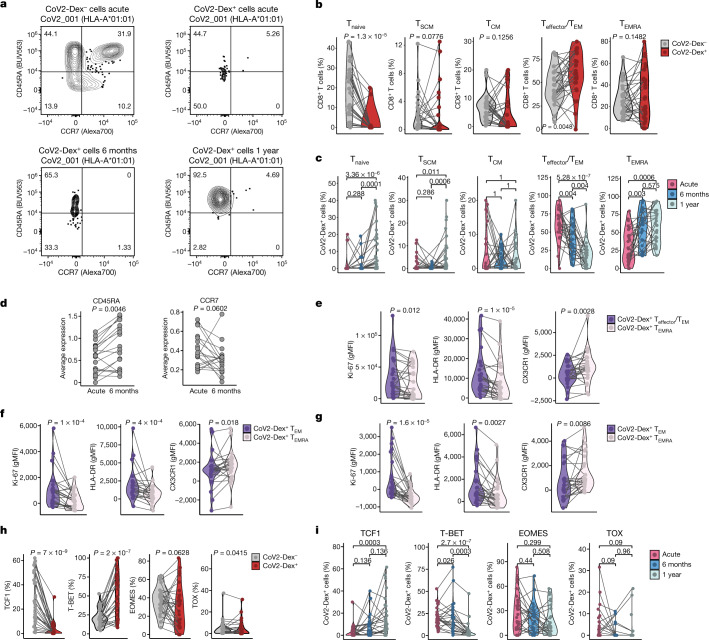
Transition of antigen-specific CD8^+^ T cells to TCF1^+^CD45RA^+^ effector memory cells at 1 year. **a**, Representative plots of CD45RA and CCR7 staining on CoV2-Dex^–^ and CoV2-Dex^+^ cells during acute infection and 6 months and 1 year after infection. Numbers in the plots indicate percentage of parent population. **b**, Percentages of T_naive_, T_SCM_, T_CM_, T_effector_/T_EM_ and T_EMRA_ cells in CoV2-Dex^–^ and CoV2-Dex^+^ cells during acute infection (*n* = 28). **c**, Percentages of T_naive_, T_SCM_, T_CM_, T_effector_/T_EM_ and T_EMRA_ CoV2-Dex^+^ cells in acute infection and 6 months and 1 year after infection (*n* = 28 acute, *n* = 24 6 months, *n* = 29 1 year). The grey lines connect individual donors sampled at different timepoints. *P* values are also shown. **d**, Expression of CD45RA (left) and CCR7 (right) determined by TotalSeq in individual CD8^+^ T cell clones in acute infection versus 6 months after infection (*n* = 41). **e**–**g**, Geometric mean fluorescence intensity (gMFI) of selected markers on T_effector_/T_EM_ and T_EMRA_ CoV2-Dex^+^ cells in acute infection (**e**), and 6 months (**f**) and 1 year (**g**) after infection. Phenotypes were evaluated only in patients with more than 5 T_effector_/T_EM_ and T_EMRA_ CoV2-Dex^+^ cells per sample (*n* = 24 acute, *n* = 24 6 months, *n* = 26 1 year). **h**, Percentages of TCF1^+^, T-BET^+^, EOMES^+^ and TOX^+^ CoV2-Dex^–^ and CoV2-Dex^+^ cells during acute infection (*n* = 28). **i**, Percentages of TCF1^+^, T-BET^+^, EOMES^+^ and TOX^+^ CoV2-Dex^+^ cells in acute infection, and 6 months and 1 year after infection (*n* = 28 acute, *n* = 24 6 months, *n* = 29 1 year). *P* values were calculated using a Wilcoxon signed-rank test in **b**, **d**–**g**, and a Wilcoxon–Mann–Whitney test with a correction for multiple comparisons using the Holm method in **c** and **i**. All tests were performed two-sided.

**Extended Data Fig. 7:**
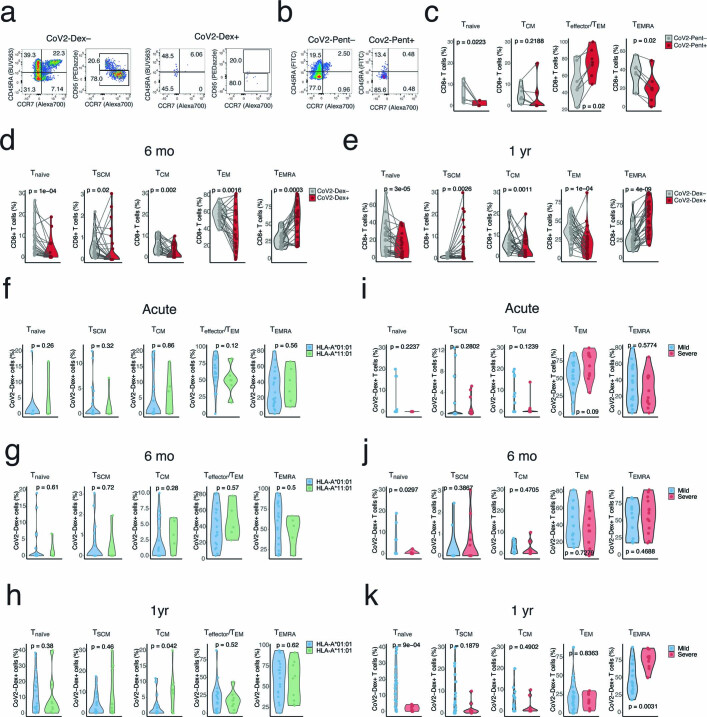
Memory phenotypes in CoV2-Dex^+^ and CoV2-Pent^+^ cells. **a**, **b**, Gating strategy for identifying naïve, stem cell memory (T_SCM_), central memory (T_CM_), effector/effector memory (T_effector_/T_EM_), and effector memory T cells re-expressing CD45RA (T_EMRA_) in (a) CoV2-Dex^–^ and CoV2-Dex^+^ cells or (b) CoV2-Pent^–^ and CoV2-Pent^+^ cells. **c**, Percentages of naïve, T_CM_, T_effector_/T_EM_, and T_EMRA_ cells among CoV2-Pent^–^ (gray) and CoV2-Pent^+^ (red) cells (*n* = 7). **d**, **e**, Percentages of naïve, T_CM_, T_effector_/T_EM_, and T_EMRA_ cells among CoV2-Dex^–^ (gray) and CoV2-Dex^+^ (red) cells (**d**) six months and (**e**) one year after infection (*n* = 24 six months, *n* = 29 one year). **f**–**h**, Percentages of naïve, T_SCM_, T_CM_, T_effector_/T_EM_, and T_EMRA_ in CoV2-Dex^+^ cells in patients with HLA-A*01:01 versus HLA-A*11:01 allele (**f**) during acute infection, (**g**) six months and (**h**) one year after infection (acute: *n* = 22 HLA-A*01:01 and *n* = 6 HLA-A*11:01; six months: *n* = 19 HLA-A*01:01 and *n* = 5 HLA-A*11:01; one year: *n* = 20 HLA-A*01:01 and *n* = 9 HLA-A*11:01). **i**–**k**, Percentages of naïve, T_SCM_, T_CM_, T_effector_/T_EM_, and T_EMRA_ in CoV2-Dex^+^ cells in patients with mild versus severe disease (**i**) during acute infection, (**j**) six months and (**k**) one year after infection (acute: *n* = 16 mild, *n* = 12 severe; six months: *n* = 11 mild, *n* = 16 severe; one year: *n* = 19 mild, *n* = 10 severe). P-values were calculated with a Wilcoxon signed-rank test in **c**–**e** and with a Mann-Whitney-Wilcoxon test in **f**–**k**. All tests were performed two-sided.

To better understand the phenotypical memory trajectories of antigen-specific CD8^+^ T cells following a naturally occurring acute virus infection, we followed CoV2-Dex^+^ cells longitudinally, at both the population level and the clonal level. In the acute phase, CoV2-Dex^+^ cells showed mostly an effector/effector memory (T_effector_/T_EM_) phenotype, whereas frequencies of naive (T_naive_) cells were lower in CoV2-Dex^+^ than in CoV2-Dex^–^ CD8^+^ T cells (Fig. 3a, b, Extended Data Fig. 7a).

These data were confirmed in CoV2-Pent^+^ cells (Extended Data Fig. 7b, c). At 6 months and 1 year after infection, we observed a progressive switch from a T_effector_/T_EM_ phenotype to a terminally differentiated T_EM_ cell re-expressing CD45RA (T_EMRA_) phenotype; thus, 1 year after infection, most CoV2-Dex^+^ cells were of a T_EMRA_ phenotype (Fig. 3c, Extended Data Fig. 7d, e). Furthermore, we observed progressive enrichment in stem cell memory T (T_SCM_) cells, particularly at the 1-year timepoint (Fig. 3c, Extended Data Fig. 7e). Of note, the increase in T_SCM_ cell enrichment was accompanied by an enrichment in T_naive_ cells 1 year after infection (Fig. 3c), possibly indicating that memory cells can reacquire CD45RA and CCR7 also in the absence of CD95 expression. We did not observe differences in memory phenotypes based on HLA, except for a tendency towards more central memory (T_CM_) cells in HLA-A*11:01 1 year after infection (Extended Data Fig. 7f–h). Conversely, memory phenotypes were strongly influenced by disease severity (Extended Data Fig. 7i–k). Patients with severe disease had fewer CoV2-Dex^+^ T_naive_ cells 6 months and 1 year after infection, tended to have fewer T_SCM_ cells and showed predominantly CoV2-Dex^+^ T_EMRA_ cells 1 year after infection. When examining individual T cell clones, we observed an increase in CD45RA expression and a concomitant decrease in CCR7 protein expression determined by TotalSeq from acute infection to 6 months after infection (Fig. 3d), thus confirming an enrichment of a T_EMRA_ phenotype also on a single TCR level.

Subsequently, we assessed whether the T_effector_/T_EM_ and T_EMRA_ phenotypes were associated with specific T cell markers, suggesting distinct differentiation states. Indeed, in CoV2-Dex^+^ cells, we observed several differences between the T_effector_/T_EM_ and T_EMRA_ populations. CoV2-Dex^+^ T_EM_ cells showed higher expression of Ki-67 and HLA-DR, whereas they had lower abundance of CX3CR1 already during acute infection (Fig. 3e). Notably, we observed the same phenotypical differences between CoV2-Dex^+^ T_EM_ and CoV2-Dex^+^ T_EMRA_ cells 6 months and 1 year after infection (Fig. 3f, g).

**Extended Data Fig. 8:**
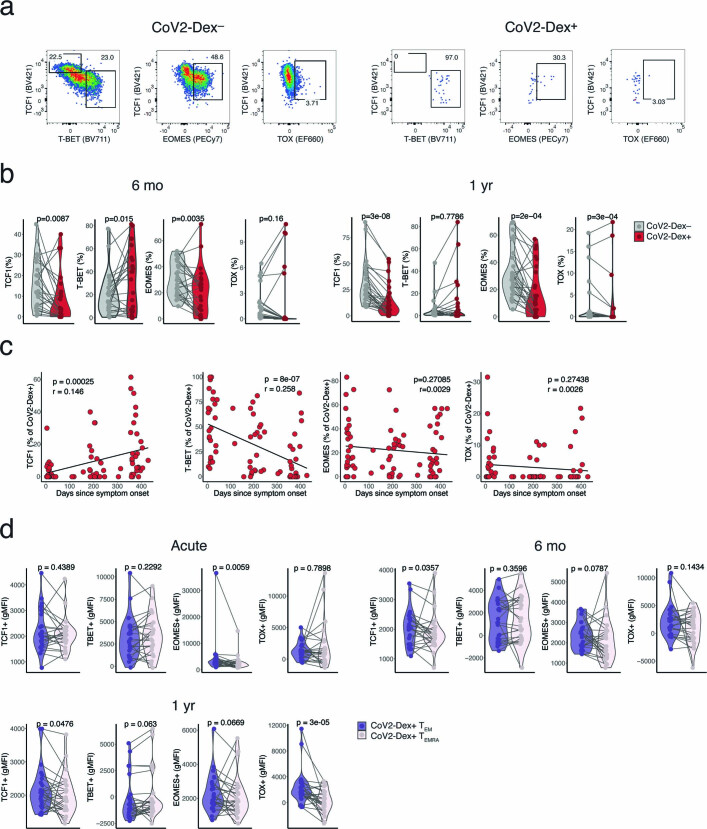
Gating strategy and pseudolongitudinal time course of transcription factor expression. **a**, Gating strategy TCF1^+^, T-BET^+^, EOMES^+^, and TOX^+^ cells in CoV2-Dex^–^ and CoV2-Dex^+^ cells. **b**, Percentages of TCF1^+^, T-BET^+^, EOMES^+^, and TOX^+^ cells in CoV2-Dex^–^ and CoV2-Dex^+^ cells (left) six months and (right) one year after infection (*n* = 24 six months, *n* = 29 one year). **c**, Linear regression of frequencies of TCF1^+^, T-BET^+^, EOMES^+^, and TOX^+^ cells in CoV2-Dex^+^ cells as a function of time since symptom onset (*n* = 81). P-values were calculated with t-statistic. **d**, Expression of transcription factors in T_effector_/T_EM_ and T_EMRA_ in CoV2-Dex^+^ cells in acute infection and six months and one year after infection (*n* = 24 acute, *n* = 24 six months, *n* = 26 one year). P-values were calculated using a Wilcoxon signed-rank test in **b** and **d**. All tests were performed two-sided.

As T cell phenotypes are driven by specific transcription factors, we assessed the expression of T cell factor 1 (TCF1), T-box expressed in T cells (T-BET), eomesodermin (EOMES) and thymocyte selection-associated high-mobility group box (TOX), which are transcription factors known to have important roles in T cell differentiation^24–27^. CoV2-Dex^+^ cells downregulated TCF1 expression during the acute phase, which was progressively restored at subsequent timepoints (Fig. 3h, i, Extended Data Fig. 8a–c). Conversely, the expression of T-BET was increased in the acute phase (Fig. 3h, Extended Data Fig. 8a) and progressively decreased 6 months and 1 year after infection (Extended Data Fig. 8b, c). A difference in T-BET expression between T_effector_/T_EM_ and T_EMRA_ CoV2-Dex^+^ cells was not evident, except for a tendency 1 year after infection. However, T_EMRA_ CoV2-Dex^+^ cells expressed lower levels of TCF1 and TOX in the memory phase and lower levels of EOMES at all timepoints (Extended Data Fig. 8d).

### Signatures of CD8^+^ memory precursors

**Fig. 4:**
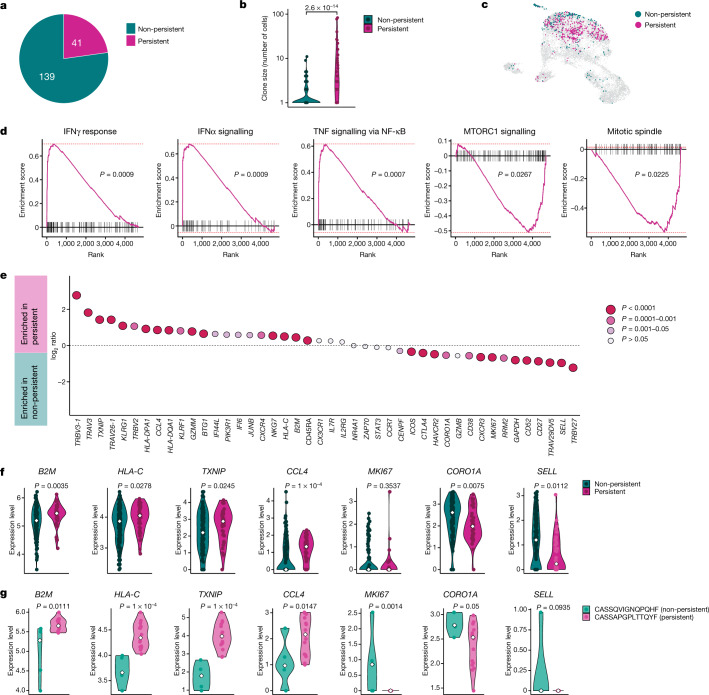
Transcriptional signature of antigen-specific CD8^+^ T cell clones persisting at 6 months. **a**, Proportion of CoV2-Dex^+^ CD8^+^ T cell clones present during acute infection that were also detectable 6 months after infection. **b**, Clone size of persisting versus non-persisting CoV2-Dex^+^ CD8^+^ T cell clones (*n* = 41 persistent, *n* = 139 non-persistent). **c**, UMAP plot of persistent (red) versus non-persistent (green) CoV2-Dex^+^CD8^+^ T cell clones detected during acute infection. **d**, Gene set enrichment analysis showing enrichment of genes associated with cytokine signalling in persistent clones and mTOR signalling and proliferation in non-persistent CoV2-Dex^+^ T cell clones. Red dashed lines indicate minimal and maximal cumulative enrichment values. *P* value calculation was performed as detailed in the Method section. **e**, Expression of selected genes and CCR7 and CD45RA protein determined by Totalseq for persistent versus non-persistent CoV2-Dex^+^ T cell clones. *P* values were calculated using a Wilcoxon signed-rank test; a Bonferroni correction was applied for multiple comparisons. **f**, Expression level of selected genes in persistent versus non-persistent individual T cell clones; each dot represents one clone. **g**, Expression level of selected genes in cells from a single non-persistent clone compared to cells from a single persistent T cell clone; each dot represents one cell (*n* = 5 CASSQVIGNQPQHF, *n* = 16 CASSAPGPLTTQYF). In **f**, **g**, the white diamonds indicate median expression. For **b**, **f**, **g**, *P* values were calculated using a Wilcoxon–Mann–Whitney test. All tests were performed two-sided.

**Extended Data Fig. 9:**
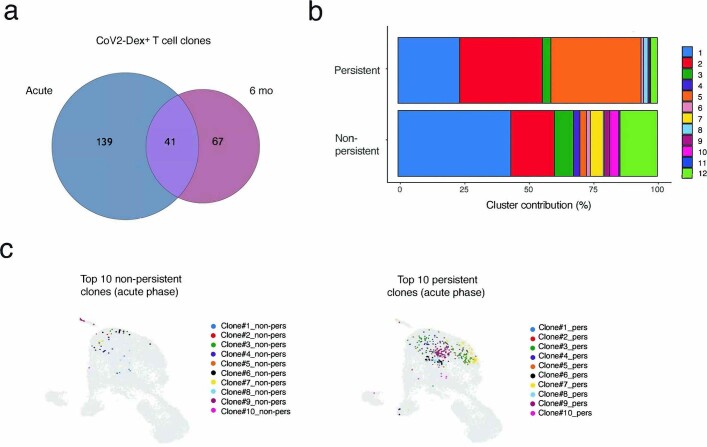
Clonality and phenotype of persistent versus non-persistent CoV2-Dex^+^ T cell clones. **a**, Venn diagram showing overlapping clones during acute infection and at six months after infection. **b**, Cluster composition of persistent versus non-persistent CD8^+^ T cell clones. **c**, UMAP plots showing top ten persistent CoV2-Dex^+^ CD8^+^ T cell clones and top ten non-persistent CoV2-Dex^+^ CD8^+^ T cell clones.

Next, we sought to identify the factors present during acute infection that instruct T cell clones towards a memory fate. We compared clones detected in the peripheral blood in both the acute and the convalescent phases (termed persistent) to those that were only seen in the acute phase and became undetectable in the convalescent phase (non-persistent) (Fig. 4a). Not all clones detected at 6 months after infection were present in the acute phase, probably reflecting a limitation of detection (Extended Data Fig. 9a). Generally, clone size correlated positively with persistence (Fig. 4b). Cells of persistent clones showed a different transcriptional makeup in the acute phase when compared to cells of non-persistent clones (Fig. 4c), which also resulted in a different distribution in the previously identified CD8^+^ T cell clusters (Extended Data Fig. 9b). This effect was robustly seen in different clones and was not due to a few hyper-expanded clones (Extended Data Fig. 9c). Gene set enrichment analysis revealed distinct signatures in persistent versus non-persistent clones. Genes involved in IFNγ and IFNα responses and TNF signalling were enriched in cells from persistent clones, whereas mechanistic target of rapamycin (mTOR) signalling and mitosis-related genes were enriched in cells from non-persistent clones (Fig. 4d). By comparing differentially regulated genes between cells from persistent and non-persistent clones, we observed genes associated with activation (*HLA-DQA1* and *HLA-DPA1*), terminal differentiation (*KLRG1*) and cytotoxicity (*GZMM* and *NKG7*), as well as certain IFN-induced (*B2M* and *HLA-C*) and TNF-induced (*CCL4*) genes to be enriched in persisters, along with CD45RA protein expression determined by TotalSeq (Fig. 4e). Conversely, cells from non-persistent clones showed higher expression of CTLA4, TIM3 (encoded by *HAVCR2*), Ki-67 (encoded by *MKI67*) and the mTOR-induced gene *CORO1A* (Fig. 4e). The same differences in gene expression could be observed at the clonal level; thus, genes were upregulated or downregulated accordingly in non-persistent individual clones compared to persistent clones (Fig. 4f), as exemplified in two selected clones of a patient (Fig. 4g). Non-persisters showed higher expression of *SELL* during the acute phase (Fig. 4e, f). We also observed differential TCR-Vβ usage between persistent and non-persistent clones (Fig. 4e).

## Discussion

In this study, we address outstanding questions related to CD8^+^ T cell memory upon acute SARS-CoV-2 infection in humans by longitudinally following individual memory CD8^+^ T cell clones. Phenotypically, we find a transition of CD8^+^ T cells from T_effector_/T_EM_ cells to T_EMRA_ cells with progressive enrichment of TCF1^+^ cells, which is paralleled by a modest enrichment in T_SCM_ cells. While two previous papers have reported a high prevalence of T_EMRA_ cells among SARS-CoV-2-specific CD8^+^ T cells^13,28^, our study provides a description of progressive enrichment in this specific phenotype during the memory phase, at both the clonal level and the population level, revealing that CD8^+^ T_EMRA_ cells might constitute the main circulating memory subset following an acute viral infection in humans.

Our data provide a different and more detailed view of individual antigen-specific human memory CD8^+^ T cells than the one observed in the tetramer-positive memory CD8^+^ T cell population in individuals vaccinated against yellow fever virus^19^, where the prevalent subset observed was T_SCM_. As that study dealt with human memory CD8^+^ T cells examined several years after vaccination, T_EMRA_ and T_SCM_ cells could be part of the same phenotypical trajectory, with progressive enrichment in T_SCM_ cells over time due to differentiation or competitive advantage. We favour an alternative hypothesis based on the phenotypical differences between memory cells in mild and severe COVID-19 that we observed, suggesting that other factors—such as antigen availability, type of antigen-presenting cells stimulated and cytokine milieu—might influence the type of memory formed, with increased T_EMRA_ cell differentiation upon severe disease versus prevalent T_SCM_ cell formation in mild disease and upon inoculation with live-attenuated virus vaccines.

We also observed enrichment of CD62L expression in CoV2-Dex^+^ cells during the acute phase rather than the recovery phase and in non-persistent clones disappearing from the circulation rather than in clones giving rise to circulating memory T cells. Whereas CD62L expression is a hallmark of recirculating T_CM_ cells^29^, which are the prevalent memory cells in lymph and secondary lymphoid organs^30^, CD62L is not typically associated with the T_EMRA_ phenotype^31^. This might explain our findings, as CD62L expression appears not to be part of the memory differentiation trajectory observed^20^. Whether a fraction of T cell clones detected in peripheral blood during the acute phase contributes to different memory pools in secondary lymphoid organs cannot be excluded in our present study.

Understanding how the immune system maintains the balance between effector response and memory formation could provide insights on why some infections result in robust and long-lasting T cell memory, whereas others fail to do so. Our study helps to unravel the complexity of these processes by finding a transcriptional signature at the level of T cell clones that correlates with the acquisition of long-lived, circulating memory T cells. We find that a strongly proliferative phenotype is associated with clonal contraction and disappearance. Furthermore mTOR signalling, probably stimulated by TCR engagement, appears to instruct the fate of short-lived effector cells, similar to previous results in mice^32^ and in vitro studies^33^. Conversely, cytokine signalling marks cells destined to become long-lived, circulating memory cells, in agreement with previous studies showing the importance of type I IFN for memory generation^34^. As we sampled SARS-CoV-2-specific T cells from the peripheral blood, we can only infer persistence of CD8^+^ T cell clones in this compartment. Importantly, memory phenotypes and the factors instructing their differentiation might vary in different immunological contexts, such as the lymph node or tissue. Similarly, as we could examine specific cells only from donors with HLA-A*01:01 and HLA-A*11:01 alleles and, to some extent, donors with an HLA-A*24:02 allele, further studies will be needed to compare our findings in other HLA types.

Collectively, our data demonstrate the formation of memory CD8^+^ T cells to be dependent on a delicate balance between cytokine and TCR signalling during acute infection, which in turn influences outcomes of long-lived, circulating memory T cells in humans.

## Methods

### Human participants and patient characteristics

Following written informed consent, adult patients with symptomatic, RT–qPCR-confirmed SARS-CoV-2 infection were recruited in the Canton of Zurich, Switzerland, between 2 April and 24 September 2020. The study was approved by the Cantonal Ethics Committee of Zurich (BASEC 2016-01440). Patients (*n* = 175) donated peripheral blood at the time of inclusion into the study, and 116 and 90 patients donated peripheral blood approximately 6 months and 1 year after infection, respectively. Standardized clinical data were collected for all included patients and disease severity was assessed, as previously described for this cohort^35–37^. Peripheral blood mononuclear cells (PBMCs) and serum were bio-banked, as previously described^35–37^. Following HLA class I typing, patients carrying an HLA-A*01:01, HLA-A*11:01 and/or an HLA-A*24:02 allele with sufficient bio-banked samples at two different timepoints were selected for the study (*n* = 47). Thirteen healthy donors carrying HLA-A*01:01, HLA-A*11:01 and/or an HLA-A*24:02 allele were included for comparison. SARS-CoV-2-specific CD8^+^ T cells were detected with MHC-I dextramers and pentamers in 42 and 12 patients, respectively.

### IgA and IgG immunoassays

Spike S1-specific IgA and IgG antibodies were assessed with a commercial ELISA kit (SARS-CoV-2 IgA and IgG immunoassay, Euroimmun), as previously described^35^. OD ratios higher than 2.0 and 1.1 were considered positive for serum IgA and IgG, respectively.

### Dextramer and pentamer staining

PBMCs (4 × 10^6^) per patient were incubated with Human TruStain FcX blocking reagent (422302, BioLegend) for 10 min at 4 °C. After washing, cells were incubated with MHC-I dextramers (see Supplementary Table 1) in the presence of l-biotin and herring sperm DNA according to the manufacturer’s instructions, for 10 min at room temperature. Two peptides presented on HLA-A*01:01 dextramers (FTSDYYQLY from ORF3a and TTDPSFLGRY from ORF1ab), two peptides presented on HLA-A*11:01 dextramers (ATEGALNTPK and KTFPPTEP from nucleocapsid protein) and one peptide presented on HLA-A*24:02 dextramers (QYIKWPWYI from spike protein) were included. For MHC-I pentamer staining, cells were incubated for 10 min at 37 °C with pentamers (Extended Data Table 1). One peptide presented on HLA-A*01:01 pentamers (FTSDYYQLY from ORF3a) and one peptide presented on HLA-A*11:01 pentamers (KTFPPTEP from nucleocapsid protein) were included. Frozen PBMCs were used throughout the study.

### Spectral flow cytometry

After dextramer or pentamer staining, a concentrated surface staining antibody mix (Supplementary Tables 2, 3) was applied without washing and samples were incubated at room temperature for further 20 min. After four rounds of washing, cells were resuspended in a fixation permeabilization solution (eBioscience Foxp3/transcription factor staining buffer) and incubated for 60 min at room temperature. After washing, an antibody mix for intracellular staining (Supplementary Tables 2, 3) was added and cells were incubated for 30 min at room temperature. After washing, samples were acquired on a Cytek Aurora spectral flow cytometer using the SpectroFlo software. Quality control for the cytometer was performed daily. Data were analysed with FlowJo (version 10.7.1) and OMIQ (www.omiq.ai). Phenotypes were evaluated only in patients with more than 5 CoV2-Dex^+^ cells per sample (*n* = 28 acute, *n* = 24 6 months after infection, *n* = 29 1 year after infection) or more than 5 CoV2-Pent^+^ cells per sample (*n* = 7 acute, *n* = 9 6 months after infection). Correlation between frequency of CoV2-Dex^+^ cells during acute infection and frequency of CoV2-Dex^+^ cells 6 months after infection was assessed only for patients sampled at least 14 days after symptom onset (*n* = 11).

### Sample sets of sorted and unsorted cells and healthy controls

A concentrated antibody mix containing TotalSeq antibodies (see Supplementary Table 4 for a complete list) was applied after dextramer staining without washing and cells were incubated at 4 °C for 30 min. After four rounds of washing, cells were resuspended in PBS with 2% FBS and 2 mM EDTA and sorted with a BD Aria cell sorter. For each patient, CoV2-Dex^–^ and CoV2-Dex^+^ cells were sorted approximately in a 10:1 ratio. All CoV2-Dex^+^ cells from each sample were sorted, the corresponding amount of CoV2-Dex^–^ cells was calculated and sorted in the same tube. Cells from ten patients at the same timepoint were pooled together, generating four individual sample sets in total: (1) patients CoV2_001–CoV2_010, acute; (2) patients CoV2_001–CoV2_010, 6 months after infection; (3) patients CoV2_011–CoV2_020, acute; and (4) patients CoV2_011–CoV2_20, 6 months after infection. Two additional sample sets were generated using 5,000 unsorted PBMCs from each patient’s sample: (5) patients CoV2_001–CoV2_010, 6 months after infection unsorted; and (6) patients CoV2_011–CoV2_020, 6 months after infection unsorted. Finally, using PBMCs from four healthy donors, we generated sample set (7) by sorting and pooling 2,000 CD8^+^ T cells from each healthy donor sample.

### scRNA-seq library preparation and sequencing

Cells of sample sets 1–7 were analysed by scRNA-seq utilizing the 5′ Single Cell GEX and VDJ v1.1 platforms (10x Genomics). Each sample set was processed individually. Cell suspensions were pelleted, resuspended and loaded into the Chromium Chip following the manufacturer’s instructions. Fourteen cycles of initial cDNA amplification were used for all sets and single-cell sequencing libraries for whole-transcriptome analysis (GEX), TCR profiling (VDJ), and combined cell-surface protein and dCODE Dextramers detection (ADT) were generated. Final libraries were quantified using a Qubit Fluorometer, pooled in a ratio of 5:1:1 (GEX:VDJ:ADT) and sequenced on a NovaSeq 6000 system with the following cycle configuration: read 1: 28 bp; index read 1: 10 bp; read 2: 101 bp.

### Single-cell transcriptome analysis

Raw scRNA-seq FASTQ files were aligned to the human GRCh38 genome with Cell Ranger version 5.0.0 with default settings for the ‘cellranger multi’ pipeline (10x Genomics). The reference genome was downloaded from the 10x Genomics website (https://cf.10xgenomics.com/supp/cell-exp/refdata-gex-GRCh38-2020-A.tar.gz) and built as per official release notes (https://support.10xgenomics.com/single-cell-gene-expression/software/release-notes/build#GRCh38_2020A). Every sample set was analysed with the ‘cellranger multi’ pipeline, which allows to process together the paired GEX, ADT and VDJ libraries for each set. Downstream analysis was conducted in R version 4.1.0 with the package Seurat version 4.0.3 (ref. ^38^). Cells with fewer than 200 or more than 2,500 detected genes and cells with more than 10% detected mitochondrial genes were excluded from the analysis.

To investigate possible patient biases, we demultiplexed cells from patient pools 1–6 based on genetic variants detected within the scRNA-seq reads. For this, we used the tool souporcell version 2 (ref. ^39^). To cluster cells based on their patient-specific genetic variants, we merged sample sets 1, 2 and 5 (comprising sorted cells from both timepoints of patients CoV2_001–CoV2_010 and unsorted cells of the same patients) and sets 3, 4 and 6 (comprising cells from both timepoints of patients CoV2_011–CoV2_020 and unsorted cells of the same patients). Then, we executed the souporcell pipeline with option *k* = 10 (the number of clusters to be determined) for each of the two merged sample sets. This analysis allowed us to classify 88% of the cells passing the filtering steps from above into 20 genotype-driven ‘patient’ clusters.

After log normalization and variable feature calculation, independent datasets were integrated using Seurat’s anchoring-based integration method. Data scaling, principal component analysis, clustering and UMAP visualizations were performed on the integrated dataset using 15 principal components and a resolution of 0.5 for the shared nearest-neighbour clustering algorithm. To define distinct biological features of cell clusters, differential gene expression analyses were done on assay ‘RNA’ of the integrated dataset. FindAllMarkers was executed with logfc.threshold and min.pct cut-offs set to 0.25. For the analysis of clusters, FindMarkers was used with default settings for the comparison of persistent and non-persistent clones. For the differential expression analysis of manually selected genes and cell-surface proteins (CD45RA and CCR7), logfc.threshold and min.pct cut-offs were set to 0.

For gene set enrichment analysis, the FindMarkers function from Seurat was first used for the differential expression of genes between cells belonging to persistent and non-persistent clones (using the default Wilcoxon rank-sum test, with options ‘min.pct=0.1, logfc.threshold = -Inf’, to account also for small expression changes, as long as the genes were expressed in at least 10% of cells of at least one group). The resulting 4,701 genes were pre-ranked in decreasing order by the negative logarithm of their *P* value, multiplied for the sign of their average log-fold change (in R, ‘-log(*P*_val)*sign(avg_log_2_FC)’). Gene set enrichment analysis^40^ was performed on this pre-ranked list using the R package FGSEA (https://github.com/ctlab/fgsea/)^41^. We used the FGSEA-simple procedure with 100,000 permutations and the hallmark gene sets for *Homo sapiens* from the Molecular Signatures Database (https://www.gsea-msigdb.org/gsea/msigdb/index.jsp, made accessible in R by the package msigdbr; https://github.com/cran/msigdbr) and set the seed value (‘set.seed(42)’ in R) before execution to make the results reproducible. For significance testing, the function fgsea::fgsea() was used, which performs a *P* value estimation based on an adaptive multi-level split Monte-Carlo scheme. A multiple hypothesis correction procedure was applied to get adjusted *P* values. The results were filtered for gene sets that were significantly enriched with adjusted *P* < 0.1.

### TCR profiling

Paired chain TCR sequences were obtained through targeted amplification of full-length V(D)J segments during library preparation. Sequence assembly and clonotype calling was done through cellranger’s immune profiling pipeline (cellranger multi). TCR profiling on filtered contig annotations was done using R package scRepertoire version 1.1.4 (ref. ^42^), which assigns TCR nucleotide and amino acid sequences together with clonal frequency counts and a clonotype classification to each cell. The function combineTCR was executed with filterMulti=T to isolate the top two expressed chains in cell barcodes with multiple chains. Clonotypes were called based on the amino acid sequence of the CDR3 region of TCRα and TCRβ chains. For cells of which only one of the two chains could be identified, the available chain was used. Clone calling was done for each sample set independently before integration.

### SARS-CoV-2 peptide-loaded dextramer binding of CD8^+^ T cells

To identify SARS-CoV-2-specific CD8^+^ T cells, we used dCODE Dextramers loaded with viral peptides presented on MHC-I molecules as described above. To assess unspecific binding, a negative control dextramer (peptide STEGGGLAY presented on HLA-A*01:01) and a general negative control dextramer were included. After analysis of the flow cytometry data, we noticed strong background staining of dextramer HLA-A*24:02 (peptide QYIKWPWYI) in samples of healthy donors, indicating unspecific dextramer binding. Thus, we excluded all sequencing counts from this dextramer in the downstream analysis. For other dextramers, cells were considered CoV2-Dex^+^ when the unique molecular identifier (UMI) count of a CoV2-dextramer was more than ten and more than five times higher than the UMI count of the negative control in the same cell. Cells that were positive for more than one dextramer according to this classification (less than 0.2% of all cells with known TCR) were excluded from the analysis. A TCR clone was considered SARS-CoV-2-specific when at least one cell of the clone was CoV2-Dex^+^.

### Statistics

Wilcoxon–Mann–Whitney test was used for comparisons of two independent groups. Wilcoxon signed-rank test was used for paired testing. *P* values were adjusted for multiple comparisons with the Holm method. A linear regression model was used to quantify the relationship between variables. Significance was assessed by non-parametric methods unless otherwise specified. All tests were performed two sided. Analyses were performed with R (version 4.0.0 or 4.1.0).

### Reporting summary

Further information on research design is available in the Nature Research Reporting Summary linked to this paper.

## Online content

Any methods, additional references, Nature Research reporting summaries, source data, extended data, supplementary information, acknowledgements, peer review information; details of author contributions and competing interests; and statements of data and code availability are available at 10.1038/s41586-021-04280-x.

### Supplementary information

Reporting Summary

Supplementary Table 1 SARS-CoV-2-specific dextramers (Dex) and pentamers (Pent).

Supplementary Table 2 Fluorophore-marked reagents used in spectral flow cytometry (dextramer staining).

Supplementary Table 3 Fluorophore-marked reagents used in spectral flow cytometry (pentamer staining).

Supplementary Table 4 Fluorophore-marked reagents used in cell sorting.

Supplementary Dataset 1 Percentages of CoV2Dex^+^ cells in CoV2Dex^+^ clones.

## Figures and Tables

**Extended Data Table 1:**
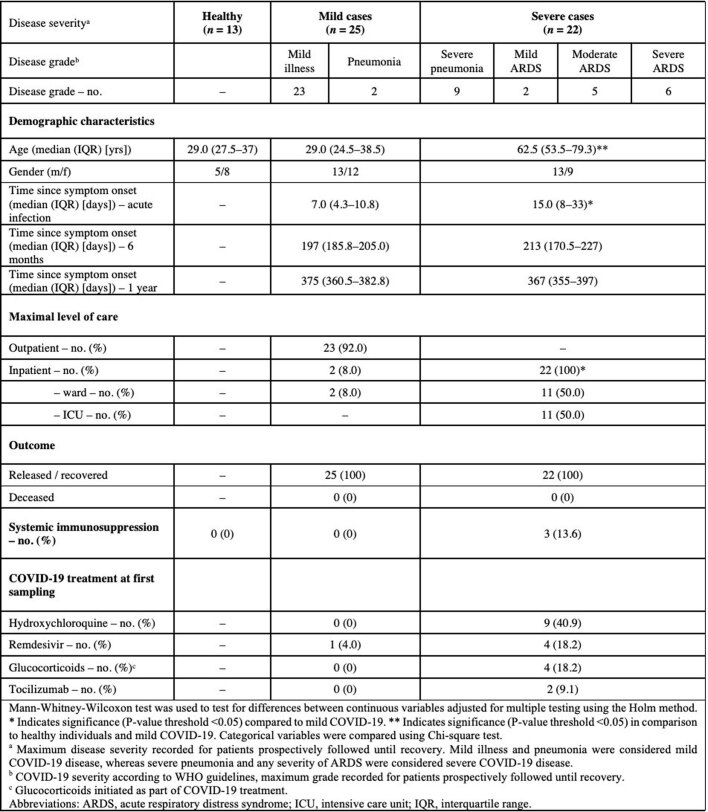
Clinical and demographic characteristics of healthy subjects and COVID-19 patients

## Data Availability

The sequencing dataset generated in this study has been deposited at zenodo.org and is available at https://zenodo.org/record/5770747. Flow cytometry datasets are available from the corresponding author on reasonable request.

## Code availability

The code generated during the current study is available at https://github.com/Moors-Code/SARS-CoV-2-Tcell-Boyman-collaboration.

## References

[1] Crotty S, Ahmed R (2004). Immunological memory in humans. Semin. Immunol..

[2] Sallusto F, Lanzavecchia A, Araki K, Ahmed R (2010). From vaccines to memory and back. Immunity.

[3] Saad-roy CM (2020). Immune life history, vaccination, and the dynamics of SARS-CoV-2 over the next 5 years. Science.

[4] Jalkanen P (2021). COVID-19 mRNA vaccine induced antibody responses against three SARS-CoV-2 variants. Nat. Commun..

[5] Chemaitelly H (2021). mRNA-1273 COVID-19 vaccine effectiveness against the B.1.1.7 and B.1.351 variants and severe COVID-19 disease in Qatar. Nat. Med..

[6] Raeber ME, Zurbuchen Y, Impellizzieri D, Boyman O (2018). The role of cytokines in T-cell memory in health and disease. Immunol. Rev..

[7] Weiskopf D (2020). Phenotype and kinetics of SARS-CoV-2-specific T cells in COVID-19 patients with acute respiratory distress syndrome. Sci. Immunol..

[8] Grifoni A (2020). Targets of T cell responses to SARS-CoV-2 coronavirus in humans with COVID-19 disease and unexposed individuals. Cell.

[9] Braun J (2020). SARS-CoV-2-reactive T cells in healthy donors and patients with COVID-19. Nature.

[10] Le Bert N (2020). SARS-CoV-2-specific T cell immunity in cases of COVID-19 and SARS, and uninfected controls. Nature.

[11] Le Bert N (2021). Highly functional virus-specific cellular immune response in asymptomatic SARS-CoV-2 infection. J. Exp. Med..

[12] Saini SK (2021). SARS-CoV-2 genome-wide T cell epitope mapping reveals immunodominance and substantial CD8^+^ T cell activation in COVID-19 patients. Sci. Immunol..

[13] Dan JM (2021). Immunological memory to SARS-CoV-2 assessed for up to 8 months after infection. Science.

[14] Zuo J (2021). Robust SARS-CoV-2-specific T cell immunity is maintained at 6 months following primary infection. Nat. Immunol..

[15] Bonifacius A (2021). COVID-19 immune signatures reveal stable antiviral T cell function despite declining humoral responses. Immunity.

[16] Hou H (2021). Immunologic memory to SARS-CoV-2 in convalescent COVID-19 patients at one-year post-infection. J. Allergy Clin. Immunol..

[17] Minervina AA (2021). Longitudinal high-throughput tcr repertoire profiling reveals the dynamics of T-cell memory formation after mild COVID-19 infection. eLife.

[18] Akondy RS (2009). The yellow fever virus vaccine induces a broad and polyfunctional human memory CD8^+^ T cell response. J. Immunol..

[19] Akondy RS (2017). Origin and differentiation of human memory CD8 T cells after vaccination. Nature.

[20] Mold JE (2021). Divergent clonal differentiation trajectories establish CD8^+^ memory T cell heterogeneity during acute viral infections in humans. Cell Rep..

[21] Graham N (2020). Rapid induction and maintenance of virus-specific CD8^+^ T_EMRA_ and CD4^+^ T_EM_ cells following protective vaccination against dengue virus challenge in humans. Front. Immunol..

[22] Peng Y (2020). Broad and strong memory CD4^+^ and CD8^+^ T cells induced by SARS-CoV-2 in UK convalescent individuals following COVID-19. Nat. Immunol..

[23] Surh CD, Sprent J (2008). Homeostasis of naive and memory T cells. Immunity.

[24] Escobar G, Mangani D, Anderson AC (2020). T cell factor 1: a master regulator of the T cell response in disease. Sci. Immunol..

[25] Intlekofer AM (2005). Effector and memory CD8^+^ T cell fate coupled by T-bet and eomesodermin. Nat. Immunol..

[26] Khan O (2019). TOX transcriptionally and epigenetically programs CD8^+^ T cell exhaustion. Nature.

[27] Wieland D (2017). TCF1^+^ hepatitis C virus-specific CD8^+^ T cells are maintained after cessation of chronic antigen stimulation. Nat. Commun..

[28] Jung JH (2021). SARS-CoV-2-specific T cell memory is sustained in COVID-19 convalescent patients for 10 months with successful development of stem cell-like memory T cells. Nat. Commun..

[29] Geginat J, Lanzavecchia A, Sallusto F (2003). Proliferation and differentiation potential of human CD8^+^ memory T-cell subsets in response to antigen or homeostatic cytokines. Blood.

[30] Buggert M (2020). The identity of human tissue-emigrant CD8^+^ T cells. Cell.

[31] Sallusto F, Lenig D, Forster R, Lipp M, Lanzavecchia A (1999). Two subsets of memory T lymphocytes with distinct homing potentials and effector functions. Nature.

[32] Araki K (2009). mTOR regulates memory CD8 T-cell differentiation. Nature.

[33] Langenkamp A (2002). T cell priming by dendritic cells: thresholds for proliferation, differentiation and death and intraclonal functional diversification. Eur. J. Immunol..

[34] Kolumam GA, Thomas S, Thompson LJ, Sprent J, Murali-Krishna K (2005). Type I interferons act directly on CD8 T cells to allow clonal expansion and memory formation in response to viral infection. J. Exp. Med..

[35] Cervia C (2021). Systemic and mucosal antibody responses specific to SARS-CoV-2 during mild versus severe COVID-19. J. Allergy Clin. Immunol..

[36] Chevrier S (2021). A distinct innate immune signature marks progression from mild to severe COVID-19. Cell Rep. Med..

[37] Adamo S (2021). Profound dysregulation of T cell homeostasis and function in patients with severe COVID‐19. Allergy.

[38] Hao Y (2021). Integrated analysis of multimodal single-cell data. Cell.

[39] Heaton H (2020). Souporcell: robust clustering of single-cell RNA-seq data by genotype without reference genotypes. Nat. Methods.

[40] Subramanian A (2005). Gene set enrichment analysis: a knowledge-based approach for interpreting genome-wide expression profiles. Proc. Natl Acad. Sci. USA.

[41] Korotkevich, G. et al. Fast gene set enrichment analysis. Preprint at 10.1101/060012 (2021).

[42] Borcherding N, Bormann NL, Kraus G (2020). scRepertoire: an R-based toolkit for single-cell immune receptor analysis. F1000Res..

