# Supplementary material for: Signature of long-lived memory CD8+ T cells in acute SARS-CoV-2 infection
Source: Nature. 2021 Dec 7;602(7895):148–55. doi: 10.1038/s41586-021-04280-x (PMC8810382; doi:10.1038/s41586-021-04280-x)
Supplement: Supplementary file 1 — Reporting Summary [file 41586_2021_4280_MOESM1_ESM.pdf]

nature portfolio

Corresponding author(s): Onur Boyman

Last updated by author(s): Nov 15, 2021

Reporting Summary

Nature Portfolio wishes to improve the reproducibility of the work that we publish. This form provides structure for consistency and transparency in reporting. For further information on Nature Portfolio policies, see our Editorial Policies and the Editorial Policy Checklist.

Statistics

For all statistical analyses, confirm that the following items are present in the figure legend, table legend, main text, or Methods section.

n/a	Confirmed	
☐	☒	The exact sample size (*n*) for each experimental group/condition, given as a discrete number and unit of measurement
☒	☐	A statement on whether measurements were taken from distinct samples or whether the same sample was measured repeatedly
☐	☒	The statistical test(s) used AND whether they are one- or two-sided<br>*Only common tests should be described solely by name; describe more complex techniques in the Methods section.*
☒	☐	A description of all covariates tested
☐	☒	A description of any assumptions or corrections, such as tests of normality and adjustment for multiple comparisons
☒	☐	A full description of the statistical parameters including central tendency (e.g. means) or other basic estimates (e.g. regression coefficient) AND variation (e.g. standard deviation) or associated estimates of uncertainty (e.g. confidence intervals)
☐	☒	For null hypothesis testing, the test statistic (e.g. *F*, *t*, *r*) with confidence intervals, effect sizes, degrees of freedom and *P* value noted<br>*Give P values as exact values whenever suitable.*
☒	☐	For Bayesian analysis, information on the choice of priors and Markov chain Monte Carlo settings
☒	☐	For hierarchical and complex designs, identification of the appropriate level for tests and full reporting of outcomes
☒	☐	Estimates of effect sizes (e.g. Cohen's *d*, Pearson's *r*), indicating how they were calculated

Our web collection on statistics for biologists contains articles on many of the points above.

Software and code

Policy information about availability of computer code

Data collection	Cytek SprectroFlo was used for acquisition of flow cytometry data. The cell Ranger pipeline (version 5.0.0) was used for acquisition of scRNAseq data.
Data analysis	FlowJo (version 10.7.0) was used for manual gating of flow cytometry data. OMIQ (Sept 2021) was used for visualization. R (version 4.0.4) was used for visualization and testing of flow cytometry data.<br>Seurat version (4.0.37) and R (version 4.1.0) was used for analysis of scRNAseq data.<br>Demultiplexing was conducted with Souporcell (version 2).<br>TCR profiling on filtered contig annotations was done using R package scRepertoire (version 1.1.4).<br>Code is available at https://github.com/TheMoorLab.

For manuscripts utilizing custom algorithms or software that are central to the research but not yet described in published literature, software must be made available to editors and reviewers. We strongly encourage code deposition in a community repository (e.g. GitHub). See the Nature Portfolio guidelines for submitting code & software for further information.

Data

Policy information about availability of data

All manuscripts must include a data availability statement. This statement should provide the following information, where applicable:

- Accession codes, unique identifiers, or web links for publicly available datasets
- A description of any restrictions on data availability
- For clinical datasets or third party data, please ensure that the statement adheres to our policy

The sequencing dataset generated during the current study is available under https://doi.org/10.5281/zenodo.5119633.
Flow cytometry datasets are available from the corresponding author on reasonable request.

Field-specific reporting

Please select the one below that is the best fit for your research. If you are not sure, read the appropriate sections before making your selection.

☒ Life sciences ☐ Behavioural & social sciences ☐ Ecological, evolutionary & environmental sciences

For a reference copy of the document with all sections, see nature.com/documents/nr-reporting-summary-flat.pdf

Life sciences study design

All studies must disclose on these points even when the disclosure is negative.

Sample size	Sample size was based on the availability of samples rather than on a pre-defined sample size calculation.
Data exclusions	Samples stained with HLA-A*24:02 dextramers were excluded from further analysis due to a strong background staining on cells from unexposed individuals. CoV2-Dex+ clones that were double positive for different dextramers were excluded from the analysis. Phenotypes were only analyzed when ≥5 cells were available.
Replication	Samples from each patient were analyzed once due to limited availability. All phenotypical and functional differences between patient groups were identified in multiple patient samples.
Randomization	In this observational study randomization was not applicable.
Blinding	Flow cytometry samples were stained in 4 batches on 4 consecutive days and in one additional fifth batch for revision. While performing the experiments the investigators were blinded to disease severity and timepoint of collection (acute, six months, one year) of the sample.

Reporting for specific materials, systems and methods

We require information from authors about some types of materials, experimental systems and methods used in many studies. Here, indicate whether each material, system or method listed is relevant to your study. If you are not sure if a list item applies to your research, read the appropriate section before selecting a response.

Materials & experimental systems

n/a	Involved in the study	
☐	☒	Antibodies
☒	☐	Eukaryotic cell lines
☒	☐	Palaeontology and archaeology
☒	☐	Animals and other organisms
☐	☒	Human research participants
☒	☐	Clinical data
☒	☐	Dual use research of concern

Methods

n/a	Involved in the study	
☒	☐	ChIP-seq
☐	☒	Flow cytometry
☒	☐	MRI-based neuroimaging

Antibodies

Antibodies used	Antibodies used for flow cytometry are listed in Supplementary Tables 1-3.
Validation	All antibodies used in this study are commercially available antibodies validated for flow cytometry analysis and/or sequencing.<br>CD4 Pacific Blue Biolegend 344620.Reactivity: Human, Application: FC, quality tested<br>CD56 BV510 Biolegend 318339. Reactivity: Human, African Green, Baboon, Cynomolgus, Rhesus. Application: FC, quality tested<br>CD3 BV785 Biolegend 300472. Reactivity: Human cross-reactivity chimpanzee, Application: FC, quality tested<br>CD8 AF488 Biolegend 344716 Reactivity: Human, Cross-Reactivity: African Green, Chimpanzee, Cynomolgus, Pigtailed Macaque, Rhesus, Sooty Mangabey, Application: FC, quality tested

CD39 APC Biolegend 328209, Reactivity: Human, Rhesus. Application: FC, quality tested
Fixable viability dye EF 780 Invitrogen 1 65-0865-14 Application: FC,
CD45RA TotalSeqTM Biolegend 304163 Reactivity: Human cross-reactivity chimpanzee, Proteogenomics, quality tested
CCR7 TotalSeqTM Biolegend 353251 Reactivity: Human, African Green, Baboon, Cynomolgus, Rhesus, Proteogenomics, quality tested
Ki-67 BUV 395 BD 564071, Reactivity: Human (QC Testing), Application: Intracellular staining (flow cytometry) (Routinely Tested)
Zombie UV UV450 Biolegend 423107, Application: FC, ICFC
CD4 BUV496 BD 564652, Reactivity: Human (QC Testing), Application: Flow cytometry (Routinely Tested)
CD45RA BUV563 BD 565703, Reactivity: Human (QC Testing), Application: Flow cytometry (Routinely Tested)
HLA-DR BUV615 BD 751142, Reactivity: Human (QC Testing), Application: Flow cytometry (Routinely Tested)
CD8 BUV661 BD 741683, Reactivity: Human (Tested in Development), Application: Flow cytometry (Qualified)
CD28 BUV737 BD 564438, Reactivity: Human (QC Testing), Application: Flow cytometry (Routinely Tested)
CD3 BUV 805 BD 612893, Reactivity: Human (QC Testing), Rhesus, Cynomolgus, Baboon (Tested in Development), Flow cytometry (Routinely Tested)
TCF-7/TCF-1 BV421 BD 566692 Reactivity: Human (QC Testing), Rhesus, Cynomolgus, Baboon (Tested in Development), Flow cytometry (Routinely Tested)
Granzyme B Pacific blue Biolegend 515408, Reactivity: Human, Mouse, Cross-Reactivity: Rat, Application: FC, ICFC
CXCR3 BV510 Biolegend 353725 , Reactivity: Human, African Green, Baboon, Cynomolgus, Rhesus, Application: FC, ICFC
PD-1 BV605 Biolegend 329923, Reactivity: Human, African Green, Baboon, Cynomolgus, Rhesus, Application: FC, ICFC
CX3CR1 BV650 Biolegend 341625, Reactivity: Human, African Green, Baboon, Chimpanzee, Common Marmoset, Cynomolgus, Rhesus, Squirrel Monkey, Application: FC - Quality tested
TBET BV711 Biolegend 644819 Reactivity:Human, Mouse, Application: ICFC - Quality tested
CD39 BV785 Biolegend 328239, Reactivity: Human, Rhesus, Application: FC - Quality tested
CD69 FITC Biolegend 310904, Reactivity: Human, African Green, Baboon, Chimpanzee, Cynomolgus, Pigtailed Macaque, Rhesus, Application: FC - Quality tested
CD57 PerCP Cy5.5 Biolegend 359621, Reactivity: Human, Application: FC - Quality tested
CD56 BB790-P BD 624296 (custom), Reactivity: Human (QC Testing), Application: Flow cytometry- Quality tested
CD95 PE Dazzle BD 562395 Reactivity: Human (QC Testing), Rhesus, Cynomolgus, Baboon (Tested in Development), Application: Flow cytometry- Quality tested
CD25 PECy5 Biolegend 302608 Reactivity: Human, Baboon, Chimpanzee, Pigtailed Macaque, Rhesus, Application: FC- Quality tested
CD127 PE-Fire 700 Biolegend 351365 Reactivity: Human, African Green, Baboon, Cynomolgus, Rhesus, Application: FC- Quality tested
EOMES PECy7 Invitrogen 25-4877-41 Reactivity: Human, Pig, Application: Flow cytometry
TOX EF 660 Invitrogen 50-6502-80 Reactivity: Human, mouse, Application: Flow cytometry
CCR7 AF700 BD 561143 Reactivity: Human (QC Testing), Application: Flow cytometry- Quality tested
TIM-3 APC fire Biolegend 345043 Reactivity: Application: FC- Quality tested

Human research participants

Policy information about studies involving human research participants

Population characteristics

Clinical characteristics of this cohort have been previously published:
Cervia, C. et al. Systemic and mucosal antibody responses specific to SARS-CoV-2 during mild versus severe COVID-19. J. Allergy Clin. Immunol. 147: 545-557.e9 (2021).
Chevrier, S. et al. A distinct innate immune signature marks progression from mild to severe COVID-19. Cell Reports Med. 2, 100166 (2021).
Adamo, S. et al. Profound dysregulation of T cell homeostasis and function in patients with severe COVID-19. Allergy 1–16 (2021) doi:10.1111/all.14866.

Age, median (IQR) [yrs]:
Healthy: 29.0 (27.5–37), Mild: 29.00 (24.50–38.50), Severe: 62.50 (53.5–79.3)

Gender m/f:
Healthy: 5/8, Mild: 13/12, Severe: 13/9

Genotypes, frequency no. (%):
HLA A01:01: Healthy: 5 (38%), Mild: 14 (56%), Severe: 14 (64%)
HLA A11:01: Healthy: 5 (38%), Mild: 8 (32%), Severe: 8 (36%)
HLA A24:02: Healthy: 3 (24%), Mild: 3 (12%), Severe: 3 (14%)

Diagnosis no. (%):
Hypertension – Mild: 0, Severe: 1 (5%)
Diabetes – Mild: 0, Severe: 0
Heart disease – Mild: 0, Severe: 1 (5%)
Lung disease – Mild: 0, Severe: 1 (5%)
Malignancy – Mild: 0, Severe: 1 (5%)
Kidney disease – Mild: 0, Severe: 1 (5%)
Cerebro-vascular disease – Mild: 0, Severe: 1 (5%)
Vascular thrombosis – Mild: 0, Severe: 1 (5%)

Treatment no. (%):
Hydroxychloroquine – Mild: 0 Severe: 9 (41%)
Remdesivir – Mild: 1 (4%) Severe: 4 (19%)
Glucocorticoids – Mild: –0 Severe: 4 (19%)
Tocilizumab – Mild: 0 Severe: 2 (9%)

Recruitment

Patients 18 years and older with symptomatic, RT-qPCR-confirmed SARS-CoV-2 infection were recruited at four different

Recruitment	hospitals in Zurich, Switzerland. Both hospitalized patients and outpatients were recruited into the study and all participants gave written informed consent. Patients had to be competent at the time of consent.<br>All out- and inpatients were recruited at the time of positive PCR test in each of the recruiting centers, if they were able to give informed consent. This introduced a potential bias as patients who were mechanically ventilated at the time of positive test could not be recruited.
Ethics oversight	The study was approved by the Cantonal Ethics Committee of Zurich (BASEC 2016-01440).

Note that full information on the approval of the study protocol must also be provided in the manuscript.

Flow Cytometry

Plots

Confirm that:

☒ The axis labels state the marker and fluorochrome used (e.g. CD4-FITC).

☒ The axis scales are clearly visible. Include numbers along axes only for bottom left plot of group (a 'group' is an analysis of identical markers).

☒ All plots are contour plots with outliers or pseudocolor plots.

☒ A numerical value for number of cells or percentage (with statistics) is provided.

Methodology

Sample preparation	Whole blood was centrifuged and plasma was removed. The remaining blood was diluted with PBS and layered into a SepMate tube (STEMCELL, catalog number 85460) filled with Lymphodex solution (Inno-Train Diagnostik GmBH, catalog number 002041500). After centrifugation, peripheral blood mononuclear cells (PBMCs) were collected and washed with PBS, resuspended in FBS with 10% DMSO and frozen.
Instrument	Cytek Aurora
Software	FlowJo (version 10.7.0) was used for gating and visualization. OMIQ was used for visualization of UMAPs.
Cell population abundance	The post sort ratio of specific to non-specific cells was checked by acquisition on a Cytek Aurora immediately after the sort for one representative donor and was approximately 1:10.
Gating strategy	Lymphocytes were gated from ungated samples (FSC-A/SSC-A), single cells were gated from lymphocytes (FSC-H, FSC-A). Live CD3+ cells were gated from single cells (CD3, live/dead). T cells were gated from CD3+ cells (CD3, CD56). CD8+ T cells were gated from CD3+ T cells. For complete gating strategy see Extended Data Figures.

☒ Tick this box to confirm that a figure exemplifying the gating strategy is provided in the Supplementary Information.
