# Supplementary material for: Signature of long-lived memory CD8+ T cells in acute SARS-CoV-2 infection
Source: Nature. 2021 Dec 7;602(7895):148–55. doi: 10.1038/s41586-021-04280-x (PMC8810382; doi:10.1038/s41586-021-04280-x)
Supplement: Supplementary file 2 — SARS-CoV-2-specific dextramers (Dex) and pentamers (Pent). [file 41586_2021_4280_MOESM2_ESM.docx]

**Supplementary Table 1. SARS-CoV-2-specific dextramers (Dex) and pentamers (Pent).**

| **Type** | **HLA allele** | **Peptide** | **Fluoro­phore** | **Nucleotide tag** | **Manu­facturer** | **Cat. #** |
| --- | --- | --- | --- | --- | --- | --- |
| Pent | A01:01 | FTSDYYQLY | PE | – | ProImmune | F4355-2A-E |
| Pent | A11:01 | KTFPPTEPK | PE | – | ProImmune | F4356B-2A-E |
| Dex | A01:01 | FTSDYYQLY | PE | AGTCGCGCAGTCTGG | Immudex | WA5973-PfBC0602 |
| Dex | A01:01 | TTDPSFLGRY | PE | TCTCTAGGTGAGTGG | Immudex | WA5974-PfBC0603 |
| Dex | A11:01 | ATEGALNTPK | PE | CAAGAACCACGCCGT | Immudex | WD5981-PfBC0605 |
| Dex | A11:01 | KTFPPTEPK | PE | CGGAATCACAATCCG | Immudex | WD5981-PfBC0606 |
| Dex | A24:02 | QYIKWPWYI | PE | AGGCGCCGTGGTGTT | Immudex | WF5952-PfBC0607 |
| Dex | A01:01 | STEGGGLAY | PE | ATACTTGTCATCTAT | Immudex | WA3579-PfBC0619 |
| Dex | General | – | PE | CCTGCACGGCCAAGC | Immudex | Ni3233-PfBC0618 |
