# Supplementary material for: Signature of long-lived memory CD8+ T cells in acute SARS-CoV-2 infection
Source: Nature. 2021 Dec 7;602(7895):148–55. doi: 10.1038/s41586-021-04280-x (PMC8810382; doi:10.1038/s41586-021-04280-x)
Supplement: Supplementary file 3 — Fluorophore-marked reagents used in spectral flow cytometry (dextramer staining). [file 41586_2021_4280_MOESM3_ESM.docx]

**Supplementary Table 2. Fluorophore-marked reagents used in spectral flow cytometry (dextramer staining).**

| **Cell marker** | **Fluorophore** | **Manufacturer** | **Cat. #** | **Dilution** |
| --- | --- | --- | --- | --- |
| CD3 | BUV805 | BD | 612893 | 1:400 |
| CD4 | BUV496 | BD | 564652 | 1:200 |
| CD8 | BUV661 | BD | 741683 | 1:200 |
| CD25 | PECy5 | Biolegend | 302608 | 1:100 |
| CD28 | BUV737 | BD | 564438 | 1:100 |
| CD39 | BV785 | Biolegend | 328239 | 1:100 |
| CD45RA | BUV563 | BD | 565703 | 1:400 |
| CD56 | BB790-P | BD | 624296 (custom) | 1:400 |
| CD57 | PerCP-Cy5.5 | Biolegend | 359621 | 1:100 |
| CD69 | FITC | Biolegend | 310904 | 1:100 |
| CD95 | PE Dazzle | BD | 562395 | 1:100 |
| CD127 | PE/Fire 700 | Biolegend | 351365 | 1:100 |
| CCR7 | Alexa700 | BD | 561143 | 1:100 |
| CXCR3 | BV510 | Biolegend | 353725 | 1:100 |
| CX3CR1 | BV650 | Biolegend | 341625 | 1:100 |
| EOMES | PECy7 | Invitrogen | 25-4877-41 | 1:100 |
| Granzyme B | Pacific Blue | Biolegend | 515408 | 1:100 |
| HLA-DR | BUV615 | BD | 751142 | 1:200 |
| Ki-67 | BUV395 | BD | 564071 | 1:100 |
| PD-1 | BV605 | Biolegend | 329923 | 1:100 |
| T-BET | BV711 | Biolegend | 644819 | 1:100 |
| TCF-7/TCF-1 | BV421 | BD | 566692 | 1:50 |
| TIM-3 | APC/Fire 750 | Biolegend | 345043 | 1:100 |
| TOX | eFluor 660 | Invitrogen | 50-6502-80 | 1:100 |
| Zombie UV | UV450 | Biolegend | 423107 | 1:400 |
