# Supplementary material for: Signature of long-lived memory CD8+ T cells in acute SARS-CoV-2 infection
Source: Nature. 2021 Dec 7;602(7895):148–55. doi: 10.1038/s41586-021-04280-x (PMC8810382; doi:10.1038/s41586-021-04280-x)
Supplement: Supplementary file 5 — Fluorophore-marked reagents used in cell sorting. [file 41586_2021_4280_MOESM5_ESM.docx]

**Supplementary Table 4. Fluorophore-marked reagents used in cell sorting.**

| **Cell marker** | **Fluorophore** | **Manufacturer** | **Cat. #** | **Dilution** |
| --- | --- | --- | --- | --- |
| CD3 | BV785 | Biolegend | 300472 | 1:100 |
| CD4 | Pacific Blue | Biolegend | 344620 | 1:100 |
| CD8 | Alexa488 | Biolegend | 344716 | 1:100 |
| CD39 | APC | Biolegend | 328209 | 1:100 |
| CD45RA | TotalSeq^TM^ | Biolegend | 304163 | 1:1000 |
| CD56 | BV510 | Biolegend | 318339 | 1:100 |
| CCR7 | TotalSeq^TM^ | Biolegend | 353251 | 1:400 |
| Fixable viability dye | eFluor 780 | Invitrogen | 65-0865-14 | 1:500 |
